# Thematic analysis of the psycho-sexual symptoms in patients with Peyronie’s disease present on online forums

**DOI:** 10.1038/s41443-022-00589-x

**Published:** 2022-06-16

**Authors:** Patrick Low, Lin Wang, Kevin D. Li, W. Patrick Shibley, Benjamin E. Cedars, Jordan T. Holler, Anthony Enriquez, Hossein Sadeghi-Nejad, Gregory M. Amend, Benjamin N. Breyer

**Affiliations:** 1 Department of Urology, University of California, San Francisco, CA USA; 2 Department of Pathology, Montefiore Medical Center, Bronx, NY USA; 3 Department of Urology, University of California, San Diego, CA USA; 4 Department of Surgery, Division of Urology, Rutgers New Jersey Medical School and Hackensack University Medical Center, Hackensack, NJ USA; 5 Department of Epidemiology and Biostatistics, University of California, San Francisco, CA USA

**Keywords:** Reproductive signs and symptoms, Erectile dysfunction, Sexual dysfunction

## Abstract

Peyronie’s disease (PD) is a fibrotic disorder of the tunica albuginea that may result in penile deformity, pain, a palpable plaque, and erectile dysfunction. In order to understand the psycho-sexual impacts of PD on patients and their partners, we selected three online forums containing the largest number of threads on PD. Threads focusing on the psycho-sexual impacts posted from January 1, 2011 to January 1, 2021 were compiled, and thematic analysis was performed on Dedoose. There were 277 unique posters, including 225 patients and 52 partners. Eighty-four categories and five themes were developed including information and social support, physical symptoms, psycho-sexual symptoms, treatment and effect, and impacts on partners and relationship. Emotional distress including depressed mood (*n* = 75, 33.3%) and feelings of isolation (*n* = 41, 18.2%) was prevalent. Partners developed sexual dysfunction including sexual dissatisfaction (*n* = 11, 21.2%) and dyspareunia (*n* = 4, 7.7%). Relationships experienced disruption (*n* = 14, 5.1%) or termination (*n* = 10, 3.6%). Posters received psychological treatment including psychotherapy (*n* = 20, 8.9%) and antidepressants (*n* = 17, 7.6%). Of these, 12 reported improvement and 11 stated no improvement. On these forums, psychological burden affecting individuals with PD and their partners is reported. Few seek help from a psychologist or therapist, and psychological distress may persist even after successful PD treatment. Further research is needed to identify strategies for effective psychological management.

## Introduction

Peyronie’s disease (PD) is a fibrotic disorder of the tunica albuginea that may result in penile deformity, pain, a palpable plaque, and erectile dysfunction. PD often presents a significant source of physical and psychological burden for both patients and their partners [1]. A previous study found that 48% of PD patients also experienced clinical depression related to their diagnosis [2]. With this degree of burden, emotional and community support can provide profound quality of life improvements [3]. However, given the sensitivity of sexual health issues, patients may be reluctant to engage with their friends, care providers, and direct community members for counseling. PD can therefore be particularly physically and mentally debilitating for many patients [3].

The psychological impact of PD on patients is well documented in the literature [4]. These studies primarily rely on questionnaires such as the Peyronie’s Disease Questionnaire (PDQ) [5] and International Index of Erectile Function (IIEF) [6]. However, the IIEF is not specifically validated for PD patients [7] and the PDQ is inherently limited by its rigid questions and requirement for patients to have recently engaged in penetrative vaginal intercourse [7]. These measures similarly do not capture the perspectives of the partners of PD patients who may also be burdened by the consequences of the disease. Furthermore, there are few qualitative studies of the impacts of PD on patients and their partners [5, 8]. There is a need for further investigation of the psychological complications caused by PD.

In this study, we aim to elucidate the psychological burden of PD on patients and their partners through first-hand accounts provided on online message boards. Recent technological developments have allowed patients to find support over online social health networks, and prior investigations have demonstrated the efficacy of internet communities in disseminating information and supporting patients [9]. They have similarly been effective in the investigation of psychological sequalae in other diseases [10]. The anonymity and support provided by these online communities provide an opportunity for PD patients to learn more about their condition and openly share their experiences without fear of embarrassment, making them ideal primary resources for investigation. Our objective is to perform a thematic analysis of PD-related online social health media forum posts. We hypothesize both PD patients and their partners will have psychosexual dysfunction resulting in decreased quality of life and relationship satisfaction.

## Materials and methods

### Forum selection

Online social health networks were identified by conducting an internet search on Google (Mountain View, CA) using the keywords “Peyronie’s disease discussion forum”. Among those identified, the three forums that contained the most publicly available threads were selected: peyroniesforum.net (https://www.peyroniesforum.net/, accessed 28 February 2021), franktalk.org (https://www.franktalk.org/, accessed 28 February 2021), and inspire.com (https://www.inspire.com/groups/peyronies-disease/, accessed 28 February 2021). Similar search phrases including “Peyronie’s disease,” “Treatment of Peyronie’s disease,” and “penile curvature” based on Google Trends data primarily returned patient education resources and subsequently were deemed inappropriate for the purposes of this investigation. Discussion boards pertaining to psychological effects of PD on patients and their partners between 2011-January 2021 were selected for analysis. Posters with more than 25% of the posts on a discussion board were excluded from our analysis to prevent bias [9]. All forum posts were publicly available and did not require registration to read. This investigation does not involve the use of human research subjects certified by the institutional review board and complies with informed consent requirements.

### Coding process

**Fig. 1:**
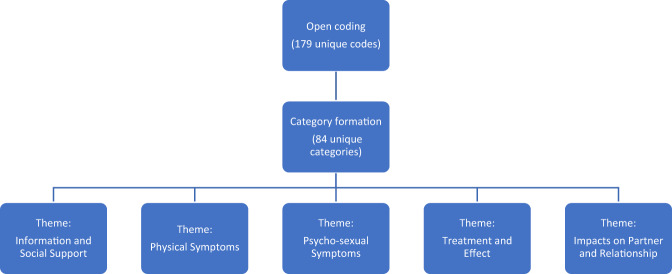
Coding Process. Thematic analysis involved a three stage process of open coding, formation of categories, and development of five major themes.

We performed a three-staged thematic analysis based on grounded theory to code the data gathered from the online message boards (Figure 1) [11]. During the initial open coding process, we analyzed our sources sentence by sentence and generated appropriate labels when the data pertained to experiences and emotions of PD patients and their partners. Additionally, we applied open codes based on interactions between posters. At this stage, open codes often took the form of in vivo codes which used terms directly from the data. An example of this is the excerpt “my penis seems so much more curved” being coded as “curvature due to PD.” The second step involved category formation in which we combined open codes into consolidated, analytic categories such as “alcohol use” and “drug use” becoming “substance use due to PD.” In the final step, we refined and sorted these categories into broader themes. A dominant category may also be used as a theme. Examples of themes include “information and social support” and “psycho-sexual concerns.” Depressive symptoms were coded if patients expressed sadness or feelings of depression independent of clinical diagnosis. Multiple codes were applied to the same excerpt whenever appropriate. Usernames of posters in each post were noted to determine the quantity of unique posters. Posters were coded as “patients” if they reported having PD or PD symptoms regardless of clinical information. Posters were coded as “partners” if they self-identified as a significant other or spouse of someone with PD.

### Analysis

All coding and descriptive analyses were conducted using the qualitative analysis software Dedoose (Los Angeles, CA. v8.3.45, 2020). Inter-rater reliability was assessed by researchers applying codes to identical posts to ensure the consistency of coding using Cohen’s Kappa coefficient. Cohen’s Kappa coefficient between coders was 0.93. Values over 0.6 regarded as substantial agreement among coders [12]. Codes involving coefficients with fair or worse agreement were discussed between coders and rectified.

## Results

There were 277 unique posters, including 225 patients and 52 partners, who contributed 853 posts. We developed 179 open codes and applied them 2543 times. Eighty-four categories were developed from the open codes. Five themes were formed from the categories: information and social support, physical symptoms, psycho-sexual symptoms, treatment and effect, and impacts on partners and relationship.

### Information and social support

Advice for other posters (*n* = 152, 54.9%) was the most common category. Due to anonymity, participants were willing to share their own experience (*n* = 87, 31.4%) and provide anecdotal knowledge (*n* = 79, 28.5%). They also often asked about the experiences of others (*n* = 71, 25.6%). Posters frequently showed gratitude (*n* = 96, 34.6%), support (*n* = 90, 32.5%), encouragement (*n* = 44, 15.9%), and sympathy and empathy towards others on the forum (*n* = 41, 14.8%) (Table 1).

**Table 1:** Information and social support.

Category	No.	%^a^
Advice for others	152	54.9
Gratitude	96	34.6
Support	90	32.5
Sharing own experience	87	31.4
Affirmation	81	29.2
Providing anecdotal knowledge	79	28.5
Inquiring about other’s experience	71	25.6
Encouragement	44	15.9
Sympathy/empathy	41	14.8
Recommending a urologist	28	10.1
Seeking support	18	6.5
Inquiring about other’s relationship	14	5.1
Inquiring about other’s partner’s experience	10	3.6
Asking for relationship advice	9	3.2
Asking for a urologist recommendation	4	1.4

^a^percentages based off total number of posters (*n* = 277).

### Physical and psycho-sexual symptoms

Curvature was the most commonly discussed physical symptom (*n* = 96, 42.7%), followed by pain (*n* = 54, 24%). Thirty-seven (16.4%) patients reported concomitant erectile dysfunction (Table 2). Emotional distress and depressive symptoms were present among patients, including depressed mood (*n* = 75, 33.3%), feelings of isolation (*n* = 41, 18.2%), and body image dysmorphia (*n* = 27, 12%), Of note, 23 (10.2%) patients expressed suicidal ideation and 15 (6.7%) patients reported developing substance use (not shown in table). Additionally, PD caused sexual distress ranging from loss of sexual confidence (*n* = 24, 10.7%) and difficulty with sex (*n* = 22, 9.8%) to avoidance of sex (*n* = 19, 8.4%) (Table 3).

**Table 2:** Physical symptoms.

Category	No.	%^a^
Curvature	96	42.7
Pain	54	24.0
Length reduction	42	18.7
Erectile dysfunction	37	16.4
Plaque	33	14.7
Girth reduction	24	10.7
Hourglassing	7	3.1
Hinging	5	2.2

^a^percentages based off total number of patients (*n* = 225).

**Table 3:** Psycho-sexual symptoms.

Category	No.	%^a^
Depressed moods	75	33.3
Feelings of isolation	41	18.2
Feelings of embarrassment	27	12.0
Anger and resentment	27	12.0
Body image dysmorphia	27	12.0
Loss of sexual confidence	24	10.7
Suicidal ideation	23	10.2
Loss of confidence	23	10.2
Nervous due to PD	22	9.8
Difficulty with sex	22	9.8
Avoidance of sex	19	8.4
Low self-esteem	15	6.7
Decreased sexual satisfaction	14	6.2
Decreased libido	13	5.8
Fear of disease progression	13	5.8
Avoidance of dating	11	4.9
Concern about further penis injury	10	4.4
Frustration toward lack of recovery	8	3.6
Feelings of inadequacy	7	3.1
Fear of rejection	3	1.3
Frustration toward disease progression	2	0.9

^a^percentages based off total number of patients (*n* = 225).

### Treatment and effect

Patients referenced non-surgical treatments including penile traction (*n* = 56, 24.9%), collagenase injection (*n* = 41, 18.2%), and vacuum pump therapy (*n* = 36, 16%). Surgical treatments included plication (*n* = 6, 2.7%), excision and grafting (*n* = 2, 0.9%), and inflatable penile prosthesis (IPP) (*n* = 2, 0.9%). Patients reported improvement in curvature after non-surgical (*n* = 17, 7.6%) and surgical (*n* = 5, 2.2%) treatment. A total of five patients reported improved confidence and sexual function after surgery. However, 12 (5.3%) patients expressed negative feelings towards the surgery they received because of lack of improvement post-operatively (*n* = 7, 3.1%), erectile dysfunction (*n* = 3, 1.3%), or loss of length (*n* = 3, 1.3%). Of those who experienced post-operative erectile dysfunction, two patients underwent incision and grafting and two had post-implant complications. Loss of length occurred after plication and lack of improvement was reported after implant by 5 patients and incision and grafting by two patients. Of note, 12 patients stated that their psychological issues were overlooked by physicians taking care of them (data not shown in table). In contrast, only a small portion received psychological treatment including psychotherapy (*n* = 20, 8.9%) and antidepressants (*n* = 17, 7.6%). Of these, 12 reported improvement and 11 stated no improvement (Table 4).

**Table 4:** Treatment and effect.

	Category	No.	%^a^
Medical/surgical therapy and effects			
	Penile traction therapy	56	24.9
	Collagenase injection	41	18.2
	PDE5 inhibitor	40	17.8
	Vacuum erection device	36	16.0
	Improvement in curvature with non-surgical treatment	17	7.6
	Negative emotions post-surgery	12	5.3
	Plication surgery	6	2.7
	Improvement in curvature with surgery	5	2.2
	Psychological improvement after treatment	4	1.8
	Loss of length after plication	3	1.3
	Erectile dysfunction post-surgery	3	1.3
	Improved sexual function after surgery	3	1.3
	Excision and grafting	2	0.9
	Inflatable penile prosthesis	2	0.9
Psychological therapy and effects			
	Psychotherapy	20	8.9
	Antidepressants	17	7.6
	Lack of improvement from therapy	11	4.9
	Improvement after antidepressants	7	3.1
	Improvement after therapy	5	2.2

^a^percentages based off total number of patients (*n* = 225).

### Impacts on partnership and relationship

Partners reported developing sexual dysfunction as well, including sexual dissatisfaction (*n* = 11, 21.2%), dyspareunia (*n* = 4, 7.7%) and decreased libido (*n* = 2, 3.8%) (Table 5). Despite communicating with and showing support to patients, a portion of partners experienced hopelessness (*n* = 11, 21.2%), feelings of being undesired (*n* = 7, 13.5%), and anxiety (*n* = 6, 11.2%). PD impacted relationships, causing relationship disruption (*n* = 14, 5.1%) or termination (*n* = 10, 3.6%). Nine couples developed alternative methods for achieving sexual satisfaction other than penetrative intercourse.

**Table 5:** Impacts on partners and relationships.

	Category	No.	%
Impacts on partners^a^			
	Sexual dissatisfaction	11	21.2
	Feelings of personal responsibility	11	21.2
	Feelings of helplessness	11	21.2
	Frustration from lack of intimacy	10	19.2
	Feelings of being undesired	7	13.5
	Anxiety	6	11.2
	Dyspareunia	4	7.7
	Anger and frustration displaced to partner	3	5.8
	Decreased libido	2	3.8
Impacts on relationship^b^			
	Difficulty communicating	15	5.4
	Relationship disruption	14	5.1
	Lack of emotional support from partners	13	4.7
	Loss of intimacy	13	4.7
	Difficulty finding a partner	11	4.0
	Relationship termination	10	3.6
	Avoidance of intimacy	9	3.2
	Development of alternative methods for achieving sexual satisfaction	9	3.2
	Concerns about not sexually satisfying partner	4	1.4

^a^percentages based off total number of partners (*n* = 52).

^b^percentages based off total number of posters (*n* = 277).

## Discussion

Contemporary studies report the prevalence of PD to be approximately 0.4–3.2% of men in the United States [13]. The psycho-sexual consequences of erectile dysfunction are frequently severe and have been well described [14, 15]. So far, there has been a paucity in the literature on the psycho-sexual impact of PD from the perspective of patients themselves. Our investigation revealed a variety of similarities and differences in comparison to the psycho-sexual morbidity described in existing literature. The major source of distress for men with PD in previous investigations was aesthetic concerns regarding the shape and size of their penis, even if function was normal. Men with PD were more distressed by the appearance of their penis than pain [16]. Similarly, our qualitative data showed that concerns about curvature were mentioned at the highest frequency of all physical PD symptoms and bothered patients the most. We speculate that curvature poses the most prominent visual deformity and therefore most frequently apparent issue for PD patients, resulting in significant adverse psychological impact more so than any other physical manifestation.

Kuja-Halkola, Henningsohn, D’Onofrio, Mills, Adolfsson, Larsson, et al. carried out the largest population based cohort study on PD to date and found that patients with PD were more likely to have psychiatric disorders including substance abuse, anxiety disorder, depression, self-injurious behavior, general psychiatric outcomes and alcohol misuse than men without PD [17]. Our investigation similarly identified the presence of substance use disorder in a subset of patients. Although substance use may act as a coping mechanism for PD patients, the presence of substance use disorder may cause further harm through additional comorbidities, psychological consequences, and increased social isolation [5]. Self-injurious behaviors are powerful predictors of suicide [18], but the percentage of patients with actual suicidal ideation was not reported by Kuja-Halkola, Henningsohn, D’Onofrio, Mills, Adolfsson, Larsson, et al. Unique to our study was the identification of PD patients with suicidal ideation. Online health forums may be valuable resources in reducing this burden as previous investigations have demonstrated the efficacy of these communities in producing long-term behavioral changes and alcohol use reduction [19]. Regardless, the excess risk of self-injurious behaviors and suicidal ideation necessitates psychological intervention and reduction of these risks stands out as an important objective for care providers. Clinicians should be aware that substance use disorder and suicide is a risk in some PD patients.

PD often impacts the sexual experience of both male and female sex partners (FSPs). The psychological consequences for FSP are often underappreciated [5, 20]. In our study, sexual partners developed sexual dysfunction including sexual dissatisfaction, dyspareunia, and decreased libido. Additionally, we found partners sometimes felt helpless, undesired, and responsible for the disease when their partners struggled with PD. We speculate that they are often left in solitude when they are not included in the evaluation and treatment plan, resulting in increased emotional distress and suffering. Female sexual dysfunction in the general population is common, estimated to impact 10–80% of women depending on age and study methodology [21–23]. Farrell, Ziegelmann, Bajic, and Levine found that that 25% of FSPs of patients with PD had evidence of sexual dysfunction based on survey responses. Referral to an expert in sexual health is warranted in these circumstances [5]. These issues may culminate in relationship disruptions and alienation of partners who often provide invaluable emotional and social support for PD patients. Additionally, our investigation found that couples developed alternative methods for achieving sexual satisfaction other than penetrative intercourse. Sex therapy may therefore promote self-acceptance and encourage couples to explore different approaches for achieving sexual satisfaction. Thus, it is important to engage both patients and partners during evaluation and treatment. Further studies should additionally investigate the impact PD on men who have sex with men. Although previous investigations have found that gay men experience greater psychosocial burden due to PD, there is a gap in the literature detailing the impacts on their male sexual partners [24].

Although psychological distress is often triggered by physical symptoms, we found that treating PD did not always resolve psychological and relationship issues. This finding is in agreement with previous investigations which have demonstrated that surgical straightening of penile curvature improved intromission comfort and penile features, but failed to improve interpersonal relationships or psychogenic ED [25]. Thus, it is important to note that depressive symptoms remained consistently high over time, suggesting PD has a lasting psychological impact [2]. For urologists, this reinforces the benefit of inquiring about psychological issues in their patients with PD and maintaining longitudinal postoperative follow up. Psychological evaluation should also be an integral component of preoperative evaluation. It helps to gauge the patient’s readiness to and expectations of surgical intervention. Any unrealistic or inappropriate expectations should be identified and addressed by the surgeon to ultimately improve patient satisfaction. Furthermore, we found that IPP and incision and grafting were associated with the highest rates of negative emotions following surgical intervention.

Among the posters affected by PD, only a portion sought or were referred to psychological intervention. This may be attributed to the fact that physicians are often unaware of the psychological sequalae of PD on patients [4]. Psychological therapy typically takes place in three different forms: individual, couple, and group therapy [3]. It may be directed at emotional, psycho-sexual, and relationship problems caused by PD. It may further take place at the time of diagnosis or follow up given the chronicity of the condition. Among patients and partners who posted about receiving therapy in any format during our investigation, the majority failed to see psychological improvements. This resistance of depressive symptoms to conventional therapy highlights the need for future investigations directed toward novel multimodal management strategies for PD associated psychological distress. It is also advisable to compare the change in psycho-sexual status of partners before and after treatment. Overall, these steps will enable clinicians to better assist couples affected by PD to overcome the complex interplay between physical and psychological burden.

Our study contained several limitations. A major limitation is the limited sample size of posters from three forums. As well, patients and their partners may suffer from a physical or psychological condition and simply not share it online. Conversely, multiple posts on different forums may have been made by the same poster. Patients and partners willing to post online may be inherently different from those who do not post, so it is unknown whether these results are generalizable to the general population. Some codes were limited in the amount of details they could capture. For example, codes such as curvature and pain due to PD do not elaborate on the severity of these symptoms. Furthermore, the qualitative design of our study is inherently subjective during the coding process and there may have been differences in code application depending on the coder. We attempted to account for this through calculation of Cohen’s kappa coefficient and thorough discussion of correct interpretation and code application. Our study design further limited analyses to descriptive statistics. Non-heterosexual men and their partners are also likely underrepresented in this analysis. Although we are able to note depressive symptoms such as sadness and self-identified depression, it is impossible to make psychiatric assessments given our methodology and these symptoms should not be taken as clinically significant diagnoses. Additionally, we analyzed posts from three particular forums that had the most publicly available information, but the opinions of these posters do not necessarily represent those of the general PD population. Evaluation of additional primary sources beyond online social health networks may capture a broader sample of PD patients.

## Conclusion

Psychological symptoms ranging from mild depression to substance abuse and suicidal ideation can be seen in patients with PD who post on online forums. PD also impacts partners and intimate relationships leading to female sexual dysfunction, psychological burden, and relationship termination. Psychological impact and distress may persist even after successful PD treatment. Few seek help from a psychologist or sex therapist. We recommend a collaborative model involving a mental health practitioner and a physician working together with the couple. Further research is needed to identify strategies for effective psychological management of PD patients.

## Data Availability

The datasets generated during and analyzed during the current study are available online at peyroniesforum.net, franktalk.org, inspire.com and from the corresponding author on reasonable request.
